# A Predictive Model for Time-to-Flowering in the Common Bean Based on QTL and Environmental Variables

**DOI:** 10.1534/g3.117.300229

**Published:** 2017-10-12

**Authors:** Mehul S. Bhakta, Salvador A. Gezan, Jose A. Clavijo Michelangeli, Melissa Carvalho, Li Zhang, James W. Jones, Kenneth J. Boote, Melanie J. Correll, James Beaver, Juan M. Osorno, Raphael Colbert, Idupulapati Rao, Stephen Beebe, Abiezer Gonzalez, Jaumer Ricaurte, C. Eduardo Vallejos

**Affiliations:** * Horticultural Sciences Department, University of Florida, Gainesville, Florida 32611; † School of Forest Resources and Conservation, University of Florida, Gainesville, Florida 32611; ‡ Agronomy Department, University of Florida, Gainesville, Florida 32511; § Agricultural and Biological Engineering Department, University of Florida, Gainesville, Florida 32611; ** Department of Agro-Environmental Sciences, University of Puerto Rico, Mayaguez, Puerto Rico 00681-9000; †† Plant Sciences Department, North Dakota State University, Fargo, North Dakota 58105; ‡‡ International Center for Tropical Agriculture (CIAT), A. A. 6713, Cali, Colombia 763533; §§ Plant Molecular and Cellular Biology Graduate Program, University of Florida, Gainesville, Florida 32611

**Keywords:** *Phaseolus vulgaris*, mixed-effects model, multi-environment trial, G × E interactions

## Abstract

The common bean is a tropical facultative short-day legume that is now grown in tropical and temperate zones. This observation underscores how domestication and modern breeding can change the adaptive phenology of a species. A key adaptive trait is the optimal timing of the transition from the vegetative to the reproductive stage. This trait is responsive to genetically controlled signal transduction pathways and local climatic cues. A comprehensive characterization of this trait can be started by assessing the quantitative contribution of the genetic and environmental factors, and their interactions. This study aimed to locate significant QTL (G) and environmental (E) factors controlling time-to-flower in the common bean, and to identify and measure G × E interactions. Phenotypic data were collected from a biparental [Andean × Mesoamerican] recombinant inbred population (F_11:14_, 188 genotypes) grown at five environmentally distinct sites. QTL analysis using a dense linkage map revealed 12 QTL, five of which showed significant interactions with the environment. Dissection of G × E interactions using a linear mixed-effect model revealed that temperature, solar radiation, and photoperiod play major roles in controlling common bean flowering time directly, and indirectly by modifying the effect of certain QTL. The model predicts flowering time across five sites with an adjusted *r*-square of 0.89 and root-mean square error of 2.52 d. The model provides the means to disentangle the environmental dependencies of complex traits, and presents an opportunity to identify *in silico* QTL allele combinations that could yield desired phenotypes under different climatic conditions.

Timing the transition from the vegetative to the reproductive stage is a key factor in defining both adaptability and successful reproduction in a given ecosystem (Worland 1996; Buckler *et al.* 2009; Li *et al.* 2015). Selective forces in play during evolution, domestication, or plant breeding aim to maximize fitness or yield (Cockram *et al.* 2007; Izawa 2007; Slotte *et al.* 2007), a major target being the time-to-flower. The timing of first anthesis depends on the developmental program governed by the genotype, and on its interactions with the environment. The major environmental factors that affect time-to-flower are photoperiod and temperature (Seaton *et al.* 2015; Song *et al.* 2013). However, the vegetative phase can be divided into an early juvenile phase and a late postjuvenile phase in which plants are first not responsive, and then responsive to environmental cues that are inductive of flowering (Cave *et al.* 2011). The duration of the juvenile phase is genetically controlled (Matsoukas 2014; Sgamma *et al.* 2014).

The mechanism that controls flowering in *Arabidopsis*, a long-day plant, is described as a highly complex network of interacting factors. This network has some built-in redundancy and it is linked to the developmental regulatory network through the action of transcription factors that act as process integrators (Posé *et al.* 2012). The allelic diversity of *Arabidopsis* flowering genes is reflected in the variation of time-to-flower among diverse ecotypes, which allowed Wilczek *et al.* (2009) to model flowering under different conditions. The genetic complexity of this trait explains why it is inherited as a quantitative trait in so many species.

In comparison to long-day plants like *Arabidopsis*, much less is known about the genetic mechanisms that regulate flowering in short day plants like soybean, rice, and maize, among others. However, some progress has been made in these species through genetic analyses that have led to the identification of several genes. For instance, QTL analysis in soybean identified several QTL known as E loci (Tasma *et al.* 2001), which determine maturity groups for this crop. Similar analyses in maize (Koester *et al.* 1993), and rice (He *et al.* 2001) have identified QTL for sensitivity to photoperiod and rate of development. Intriguingly, more detailed molecular genetics analyses have revealed that several QTL associated with time-to-flower in short-day species are orthologs of genes that regulate flowering in *Arabidopsis* (Simpson and Dean 2002; Lee and An 2007; Salomé *et al.* 2011). For example, rice QTL *HD1* and *HD3a* were found to be orthologs of *Arabidopsis* Constans and *FT*, respectively (Kojima *et al.* 2002; Tamaki *et al.* 2007). These analyses have also discovered some flowering QTL that do not have an ortholog in *Arabidopsis*, like the *EARLY HEADING DATE 1* QTL of rice (*Ehd1*), a gene that regulates the expression of *FT* (Itoh *et al.* 2010). This finding suggests that additional mechanisms that control time-to-flower are likely to be discovered in short-day plants. Emphasizing this point is the discovery that *Setaria*, a short-day grass, contains a secondary mechanism that operates under long days (Doust *et al.* 2017).

The common bean (*Phaseolus vulgaris* L.) is a facultative short-day plant (Garner and Allard 1920). Wild bean accessions, as well as most Andean cultivars, are mainly photoperiod sensitive (short-day response), whereas Mesoamerican cultivars are mostly less sensitive to photoperiod, or day-neutral (White and Laing 1989). The prevalence of photoperiod insensitivity among the most widely cultivated beans indicates that day-neutrality in beans is a recently acquired trait, most likely the result of selection pressure applied during domestication and more recent breeding efforts. White and Laing (1989) used the observed difference in days-to-flower between plants grown under 12.5 and 18 hr photoperiods to identify eight photoperiod response classes (Classes 1–8) in this species. Under the 18 hr day-length regime Class 1 displayed 0–3 d delays, while Class 8 displayed over 100 d delays. Bean cultivars belonging to these Classes are cultivated in a variety of conditions around the world (Beebe 2012). In the United States, the common bean is cultivated as a summer crop under long photoperiods in the Northern plains, and as a winter crop under short days in Florida. The genetic manipulation of the time-to-flower trait is one of the major targets in plant breeding programs, and the wide geographical range of cultivation obviously presents a major challenge.

Previous investigations have identified a strong dominant photoperiod sensitive (*Ppd*) gene regulating flowering time in beans (White and Laing 1989; Wallace *et al.* 1991; Kornegay *et al.* 1993; White *et al.* 1996; Gu *et al.* 1998; Kwak *et al.* 2008), but a rigorous genetic analysis of this trait has not been carried out. In the present study, we used a multi-environment mixed-effects model, as described by Malosetti *et al.* (2013), to analyze the contribution of genetic (QTL), environmental, and QTL-by-environment interactions factors to the time-to-flower trait in an intergene pool recombinant inbred family of the common bean. The main aim of this study was to develop a QTL-based environmental predictive model capable of estimating time to flowering (TF) of a bean plant based on its genotype and environmental conditions in which it is grown.

## Materials and Methods

### Mapping population

A recombinant inbred (RI) population was generated from a biparental cross between the Mesoamerican bean cultivar Jamapa and the Andean cultivar Calima. F_2_ seeds of the cross were advanced by single seed descent for 10 generations, followed by bulk propagation for another three generations, giving rise to 188 F_11:14_ RI lines. Jamapa is a small black seeded (*c*) (Prakken 1974) bean cultivar from Mexico with an indeterminate growth habit (*Fin*, *pvTFL1y*) (Repinski *et al.* 2012). The parental line Calima from Colombia is a large-seeded mottled bean cultivar (*C*), with determinate (*fin*) growth habit. White and Laing (1989) classified Jamapa as a day-neutral variety (Class 1), while Calima was reported to be a photoperiod-sensitive cultivar (Class 5). Long days (18 hr day length) delay flowering of Class 1 genotypes by 0–3 d, while Class 5 genotypes are delayed by 40–59 d (White and Laing 1989).

### Experimental sites

Five distinct locations providing contrasting growing conditions were selected to phenotype the RI population for time to first flower after planting (Supplemental Material, Figure S1 in File S1 and Table 1). Three sites were located in the United States: Citra, FL (CIT); Prosper, North Dakota (ND); and Isabela, Puerto Rico (PR), while the remaining two sites were in Colombia: Palmira, (PAL), and Popayan, (POP). Proximity of PAL and POP to the equator provided short days, whereas altitudinal difference (800 m) resulted in a temperature differential (Table 1). PAL and PR had similar photoperiod and temperature range, but differed in solar radiation. Situated farthest away from the equator, ND provided long days (15:20–15:53 hr) from sowing to first anthesis, and CIT provided high temperatures and intermediate photoperiod length.

**Table 1:** Experimental sites

Location	Site	Latitude	Longitude	MASL*^a^*	Temperature (°)*^b^*	Solar Rad*^c^* (MJ m^−2^ d^−1^)	Day-Length Range (hr)
Citra, FL	CIT	29° 39’ N	82° 06’ W	31	32/18	20.6	12:30–13:30
Prosper, ND	ND	47° 00’ N	96° 47’ W	280	27/13	21.0	15:20–15:53
Palmira, Colombia	PAL	03° 29’ N	76° 81’ W	1000	28/19	13.8	11:56–11:58
Popayan, Colombia	POP	02° 25’ N	76° 62’ W	1800	25/13	15.0	12:08–12:11
Isabela, PR	PR	18° 28’ N	61° 02’ W	128	29/19	21.5	11:30–12:35

Geographical data and meteorological characteristic recorded during the growing season.

a Meters above sea level.

b Average high/low.

c Daily average solar radiation.

Primary trials were conducted between 2011 and 2012, and one trial was conducted per site (Table S1 in File S1). An additional trial in 2016 (CIT_16) was conducted at the CIT site to generate a dataset for model validation (Table S1 in File S1). The plant density at each site was adjusted based on available resources (Table S1 in File S1). A randomized complete block row-column design was adopted at each site. A given recombinant inbred line (RIL) was sown in three replicated plots at each site, with 35–50 plants per plot. Parental lines were, however, replicated six times to provide additional checks. Six uniform plants per plot were tagged at the V1 (first trifoliate opening) stage for collection of phenological data, giving 18 observations per genotype per site. The tagged plants were monitored daily to record the date at which first anthesis was observed. TF (days) for a given plant was defined as the number of calendar days it took to first flower from the date of sowing. The mean of six plants per plot was utilized for further analysis. Along with flowering time, data related to additional 30 phenotypic traits were also collected at each location, but not used in this analysis.

### Heritability

TF data from each of the five primary locations were spatially corrected to reduce the noise caused by within field variation. A linear mixed-effect model was constructed to obtain adjusted mean predictions of the following form:

$${\text{TF}}_{\mathrm{ijkl}}=\mu +{\text{G}}_{i}+{\text{B}}_{j}+{\underline{\text{BR}}}_{\mathrm{jk}}+{\underline{\text{BC}}}_{\mathrm{jl}}+{\underline{\varepsilon }}_{\mathrm{ijkl}}$$ (1)

where μ = population mean, G*_i_* = *i*th genotype effect, B*_j_* = *j*th replication effect, BR*_jk_* = effect of the *k*th row within the *j*th replication, BC*_jl_* = effect of the *i*th column within the *j*th replication, and ε*_ijkl_* = residual error. The underlined terms represent random effects, while the remaining terms were treated as fixed effects in the model. Furthermore, genetic correlations were assumed to be 0, 0.5, and 0.5 between the parental lines, between a parent and a RI line, and among RILs, respectively.

Broad-sense heritability (H^2^) of the TF trait was obtained in two ways: (i) using individual site data, and (ii) using all five sites data. First, site-based calculations were carried out by refitting the genotype (G*_i_*), and replication (B*_j_*) terms as random effect in Equation 1. The heritability was estimated by utilizing the variance component information obtained from the model using the equation H_s_^2^ = Var(G)/Var(P), where the heritability (H_s_^2^) is represented as the fraction of the total phenotypic variance Var(P), explained by the genetic variance Var(G). Second, a multi-site mixed effect model (Equation 2) incorporating correlations among the sites was constructed in ASReml (Gilmour *et al.* 2009) to re-estimate broad sense heritability at the individual site-level (H_R_^2^) as well as combined heritability using all five sites (H_T_^2^). The fitted model had the following form:

$${\text{TF}}_{\mathrm{ijklm}}=\mu +{\text{S}}_{m}+{\text{SB}}_{\mathrm{mj}}+{\underline{\text{SG}}}_{\mathrm{mi}}+{\underline{\text{SBR}}}_{\mathrm{mjki}}+{\underline{\text{SBC}}}_{\mathrm{mjl}}+{\underline{\varepsilon }}_{\mathrm{ijklm}}$$ (2)

where μ = population mean, S*_m_* = effect of the *m*th site, SB*_mj_* = interaction between the *j*th replication and the *m*th site, SG*_mi_* = nested effect of the *i*th genotype in the *m*th site, SBR*_mjk_* = effect of the *k*th row within the *j*th replication at the *m*th site, SBC*_mjl_* = effect of column within the *j*th replication at the *m*th site, and ε*_ijkl_* = residual error. As before, the underlined terms are random effects.

The variance–covariance component was modeled using an unstructured variance (UN) matrix for the term SG*_mi_*. The heritability at each site (H_R_^2^) was obtained by dividing the variance due to genotype × site with the total variance (*i.e.*, summed variance due to the effect of row, column, genotype × site and the error term). The overall trait heritability (H_T_^2^) across sites was calculated by taking the average of the variance due to genotype × site across the five primary locations, and dividing it by the average of total variance across five sites.

### Genotypic data

The mapping population comprised of 188 RI lines that were genotyped earlier (Bhakta *et al.* 2015) using the genotyping-by-sequencing (GBS) (Elshire *et al.* 2011) protocol. The GBS-linkage map included 513 recombinationally unique markers comprising 11 linkage groups (Bhakta *et al.* 2015). The map had on an average one marker per 1.84 cM, and alignment of this map with the available *P. vulgaris* reference genome sequence (www.phytozome.net) revealed a genome coverage of >97%. The genotypic data of this RI family, obtained by Bhakta *et al.* (2015), were recoded for each RIL as −1 or +1 representing homozygous loci for Jamapa or Calima alleles, respectively. No heterozygous markers were considered. Missing markers information was imputed within the GenStat v.17 (Payne *et al.* 2010) software using a hidden Markov model as described by Lander and Green (1987).

### Multi-environment QTL mapping

QTL controlling TF in the RI population were identified independently by (i) GenStat v.17 (Payne *et al.* 2010), which uses a mixed effect model approach; and (ii) WinQTL Cartographer (Wang *et al.* 2012), which utilizes various interval mapping approaches. The phenotypic response (TF) for QTL analysis for a given genotype at a given primary site was computed by averaging the adjusted mean data from all three replications, giving one value of TF per genotype per site.

The initial step for mapping QTL using GenStat was to identify the best variance-covariance matrix model for the phenotypic data (Malosetti *et al.* 2013). Subsequently, simple interval mapping (SIM) was carried out to perform a preliminary scan of the genome for mapping QTL using the 513 markers (genetic predictors). The identified QTL were used as cofactors in a follow up composite interval mapping (CIM), which allowed reduction of the background noise due to QTL outside the genomic region under test. QTL scanning was performed with a window size of 5 cM, while a 50 cM distance was used as the minimum cofactor distance in the CIM scan. For both SIM and CIM the *P*-value threshold value for detection of significant QTL was computed using a modified Bonferroni correction method as described by Li and Ji (2005). Using a genome-wide significance level of 0.05, the software estimated the threshold value of 3.447 (−log10P) for detecting QTL governing the trait TF.

QTL identified through CIM were simultaneously incorporated into a mixed-effect model with the appropriate variance-covariance matrix identified for the TF trait. Significance of each QTL was tested based on the Wald test statistic, and the final model was selected using backward selection based on the Akaike’s Information Criterion (AIC; Akaike 1974). The QTL-based mixed-effect model contained main individual QTL effects and QTL × Site interaction effects allowing to explain genotype-by-environment interactions (GEI). The mixed-effect QTL model had the form:

$${\text{TF}}_{\mathrm{ij}}=\mu +{\text{G}}_{i}+{\text{S}}_{j}+\sum \left(\alpha {\text{X}}_{\mathrm{iq}}\right)+\sum \left(\beta {\text{X}}_{\mathrm{iq}}{\text{S}}_{j}\right)+\varepsilon_{\mathrm{ijk}}$$ (3)

where μ = population mean, G*_i_* = random effect of the *i*th genotype, S*_j_* = effect of the *j*th primary Site, α = effect of the *q*th QTL on TF, X*_iq_* = marker value of −1 (Jamapa) or 1 (Calima) at the *q*th QTL for *i*th RIL, β = effect on TF due to the interaction between the *q*th QTL and the *j*th Site, and ε*_ijkl_* = residual error. The “G” term captured the genetic effect on TF not explained by the identified QTL.

Additional QTL analyses were performed using WinQTL Cartographer. Here, an initial scan was performed using CIM with standard model settings and forward and backward regression using a 5-cM window size. The CIM output was subsequently used to perform multiple interval mapping (MIM; Zeng *et al.* 1999), in order to identify significant QTL as well as epistatic interactions among identified QTL. Empirical thresholds corresponding to 0.05 genome-wide significance levels were computed for CIM likelihood ratio tests based on 1000 permutations. Unlike GenStat, WinQTL Cartographer performed QTL analysis for each environment separately.

### Modeling the TF trait

The above-mentioned model (Equation 3) provided the basis for predicting TF for each RIL. However, in order to build the predictive model, the Site term “S*_j_*” was broken down into informative measurable environmental variables. These variables were estimated to have influence on flowering time at a given site, both directly and/or through their interaction with specific QTL. To identify significant environmental variables, we selected those that are known to have an effect on the TF in *Arabidopsis* (Somers *et al.* 1998; Devlin and Kay 2000; Suárez-López *et al.* 2001; Thomas *et al.* 2006; Lee *et al.* 2007; Strasser *et al.* 2009) and rice (Kojima *et al.* 2002; Tamaki *et al.* 2007; Ishikawa *et al.* 2011; Itoh *et al.* 2010). These variables were classified into two categories: (1) Light related: Day-length (DAY, hr), night duration (NIGHT, hr), and average daily solar radiation (Srad, MJ m^−2^ d^−1^); and (2) temperature related: minimum temperature (Tmin, °), average temperature (Tavg, °), maximum temperature (Tmax, °), average day time temperature (DTavg, °), and average night time temperature (NTavg, °). Later, RIL-specific data for these variables were averaged for the duration observed between the sowing date and the date at which the given RIL flowered at a given site. Successively, correlations among the environmental covariates (EC) were calculated using the Spearman’s rank correlation coefficient test allowing us to remove redundant variables.

ECs found with Spearman’s ρ < 0.5 were selected for modeling and were sequentially incorporated into the QTL × Site term by replacing the “Site” term in order to test their interaction with a given environmentally guided QTL. Subsequently, the main effects of ECs were added to the model. The significance of each of the new terms was statistically tested with the assumption of a linear relationship between TF, and QTL and ECs effects. Selection of significant terms was based on Wald test statistics (*α* = 0.05), which were generated by fitting the Equation 3 model in GenStat (Payne *et al.* 2010; Malosetti *et al.* 2013). In the final model, the site term was replaced with significant ECs, while the GEI terms were replaced with QTL × ECs interactions. Therefore, the final model was tested for its ability to predict TF based on specific climatic data and QTL information.

The parameter estimation process for all models was carried out using the flowering data from the five primary locations. Parental data were not utilized during the process of coefficient estimation. Model evaluation was conducted in three parts: (i) by estimating flowering time of each parental genotype at all five primary locations; (ii) by estimating flowering time of 100 recombinant inbred lines regrown in 2016 at the CIT location (CIT_16); and (iii) by estimating parameters using data from only four primary sites, and then estimating flowering time for the genotypes grown at the fifth site.

### Data availability

Genotype data and mapping information from the RIL family can be found in Bhakta *et al.* (2015). The environmental data are described in Figure S1 and Table S1 in File S1 and Table 1. The days-to-flowering data are available in Figshare at https://figshare.com/s/50d1ddcaf8c04026dd4c. Seeds of the parental genotypes and RILs are available upon request.

## Results

The RIL population, generated from an intergene pool cross between the indeterminate Mesoamerican cultivar Jamapa and the Andean determinate cultivar Calima, was grown and phenotyped for TF at the five distinct sites listed in Table 1 (see also Figure S1 and Table S1 in File S1).

### Time to first anthesis

**Figure 1:**
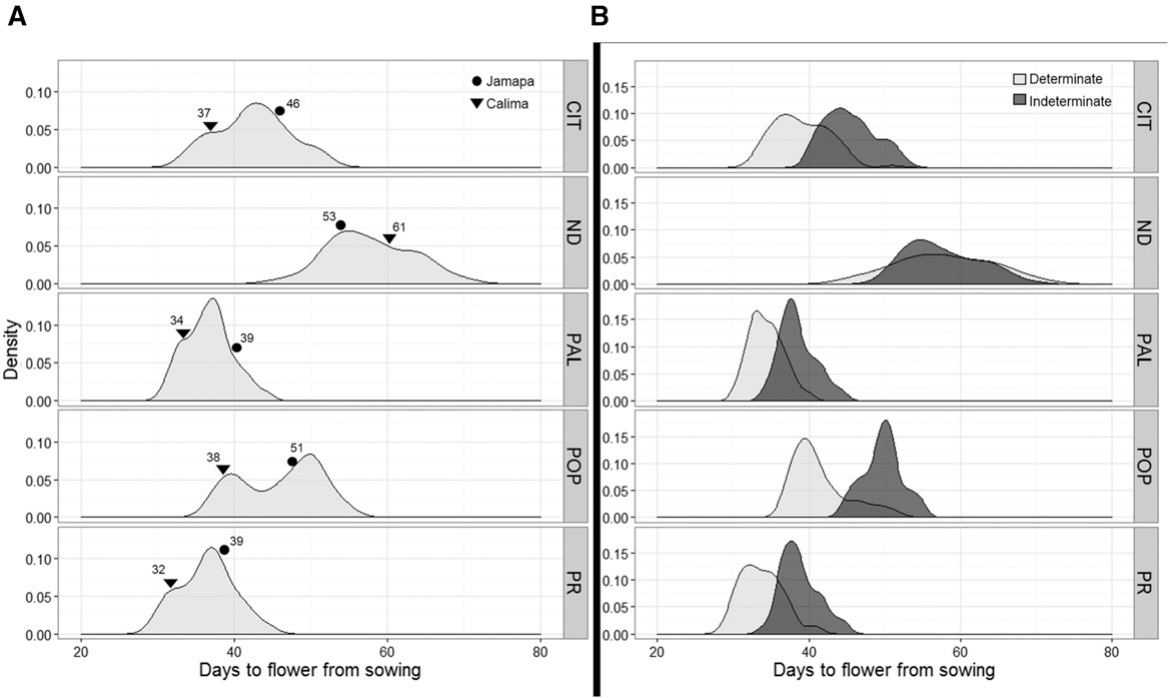
Density plots of days to first flower observed at the five experimental sites. (A) Distribution of days to flower of the RI family by site. (B) Distribution of days to flower across five sites based on growth habit; the light gray areas represent the distribution of determinate RI lines, while dark gray areas represent the indeterminate RI lines. The five sites include: Citra, FL (CIT); Prosper, North Dakota (ND); Palmira, Colombia (PAL); Popayan, Colombia (POP); and Puerto Rico (PR).

TF in the RIL population was significantly affected by the genotype and by the environment (Table S2 in File S1). Based on Bonferroni’s comparison tests, CIT, ND, and POP had significantly (*P* = 0.05) different TF (Table S3 in File S1), and these sites were also different from PR and PAL (Table S3 in File S1). TF was most delayed at the ND site with a mean of 57.8 d, whereas the shortest average TFs were detected at PR and PAL, each with 36.4 and 36.7 d, respectively (Table S3 in File S1). TF had a near bell-shaped distribution across all sites, except at POP where it displayed a strong bimodal distribution (Figure 1A). Jamapa flowered later than Calima at CIT, PAL, PR, and POP; while the opposite was true at the ND site (Figure 1A). Transgressive behavior was detected at all sites as several RILs flowered earlier or later than the parental lines. On average, the group with an indeterminate growth habit flowered later than the determinate group at all but the ND site (Figure 1B); however, the length of the delay was site dependent.

The heritability of the TF trait was first calculated in order to assess the magnitude of its genetic component. Accordingly, broad-sense heritability of the TF trait was estimated using two separate models: (i) a site-specific linear mixed-effect model, and (ii) a multi-site mixed effect model capturing inter-site correlations. According to the single-site based model (See *Materials and Methods*, Heritability) the estimated broad-sense heritabilities (*H_S_*^2^) ranged from 0.69 at ND to 0.89 at POP and PAL (Table S4 in File S1). Similar site-specific heritability (*H_R_*^2^) estimates (Table 2) were obtained from the multi-site model (See *Materials and Methods*, Heritability). This model also allowed estimation of the overall TF heritability (*H_T_*^2^), which was calculated to be 0.78 (Table 2). These results clearly indicated that the TF trait is under strong genetic control in all environments.

**Table 2:** Site level *H_R_*^2^ and *H_T_*^2^ for TF estimated using a multi-site mixed-effect model

	*H_R_*^2^					*H_T_*^2^
Component	PR	ND	POP	CIT	PAL	Overall
Rep × Row	0.244	1.449	0.044	0.189	0.000	0.385
Rep × Col	0.572	0.522	0.542	0.383	0.165	0.437
Site × RIL	12.846	36.015	25.323	21.148	8.691	20.805
Error	2.939	14.227	2.596	3.543	0.907	4.842
Total	16.600	52.212	28.505	25.263	9.763	26.469
Heritability	0.774	0.690	0.888	0.837	0.890	0.786

Estimations were made for the RI population grown at the five sites: PR, ND, POP, CIT, and PAL.

### TF in the common bean is under polygenic control

**Figure 2:**
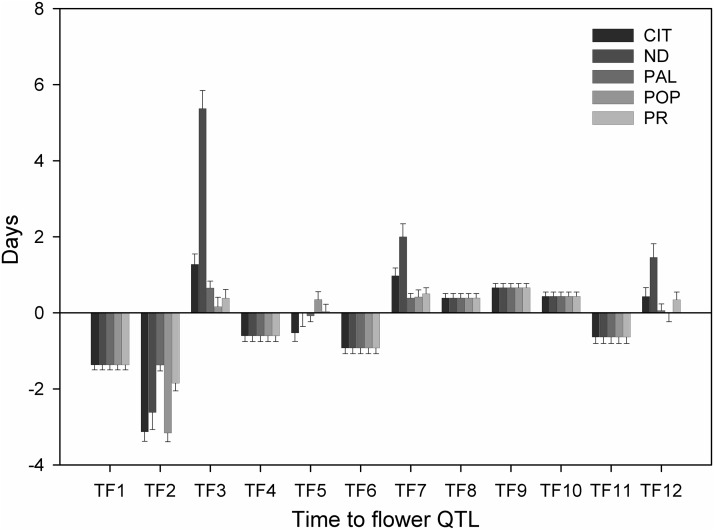
Effects of TF QTL (TF1: TF12) as estimated by the mixed-effect model across five locations. Bar height indicates the number of days a QTL homozygous for the Calima allele will add to or subtract from the population mean of the TF trait. The five sites include: CIT, ND, PAL, POP, and PR.

The continuous distribution of TF (Figure 1) underscored the quantitative nature of this trait, and its high heritability value indicated the feasibility of identifying important genetic factors controlling it. Furthermore, the transgressive behavior of some RILs and the marked change in the trait distribution between the experimental sites suggested TF is a complex trait with noticeable GEI effects. Consequently, QTL analyses were carried out to identify the genetic determinants of this trait and their mode of action. Following an evaluation of various covariance models using the AIC (Akaike 1974), the unstructured variance-covariance model of the phenotypic data were identified as the best for studying QTL effects in our RIL population (Table S5 in File S1). With this information, along with 513 marker loci, SIM, CIM, and mixed-effect modeling were used sequentially in GenStat v.17 (Payne *et al.* 2010) to identify QTL that exerted significant control of TF. These analyses identified 12 significant QTL (named TF1–TF12) distributed along six chromosomes (Chr): 1, 3, 4, 6, 7, and 11 (Table 3). Furthermore, site-based QTL effects estimated via the mixed-effect model revealed that TF2 (Chr1) explained most of the genetic variation at CIT (35.4%) and POP (37.2%) (Figure 2 and Table S6 in File S1), whereas TF3 (Chr1) explained 39% of the variation in ND.

**Table 3:** QTL detected for TF using a multi-site mixed-effect model

QTL ID	Linked Marker*^a^*	Chromosome	Position (cM)	−log10(P)	QTL × Site
TF1	DiM_1-13	1	22.1	23.90	No
TF2	Fin	1	42.1	49.82	Yes
TF3	DiM_1-28	1	58.8	28.95	Yes
TF4	DiM_1-34	1	70.0	4.22	No
TF5	DiM_3-22	3	38.2	2.83	Yes
TF6	DiM_3-27	3	49.2	8.77	No
TF7	DiM_4-13	4	42.2	8.39	Yes
TF8	DiM_6-22	6	31.3	3.21	No
TF9	Bng249	7	11.7	8.90	No
TF10	DiM_7-39	7	98.7	3.91	No
TF11	Bng076	11	2.1	3.80	No
TF12	DiM_11-2	11	9.3	3.70	Yes

Five out of 11 QTL were found to interact with the environment.

a For marker name and position please refer to Bhakta *et al.* (2015).

Both parents were found to have QTL alleles that either delayed or hastened the time to flower. These explained the transgressive behavior observed in the RIL population. For instance, Calima alleles of TF1, TF2, TF4, TF6, and TF11 reduced flowering time (TF), while alleles of TF3, TF7, TF8, TF9, TF10, and TF12 increased time to flower (Figure 2); whereas the Jamapa alleles had the opposite effect. Interestingly, the TF5 Calima allele delayed TF at POP, but reduced it at CIT. Out of the 12 QTL, five (TF2, TF3, TF5, TF7, and TF12) were found to interact with the environment, and the rest were stable across environments (Figure 2 and Table 4). For example, the Calima allele of TF1 identified as environmentally stable, reduced TF at all sites by ∼1.4 d. In contrast, the Calima allele of TF2 displayed significant interactions with the environment, and reduced TF at CIT, ND, PAL, POP, and PR by ∼3.1, 2.6, 1.3, 3.1, and 1.8 d, respectively (Table S6 in File S1).

**Table 4:** Conditional Wald statistic tests for significant fixed effects of the TF predictive model incorporating QTL × Site effects

Fixed Term	Wald Statistic	Degrees of Freedom (d.f.)	Wald/d.f.	*P*-Value
SITE	8353.76	4	2088.44	<0.001
TF1	113.57	1	113.57	<0.001
TF4	15.93	1	15.93	<0.001
TF6	43.4	1	43.4	<0.001
TF8	12.07	1	12.07	<0.001
TF9	39.83	1	39.83	<0.001
TF10	12.63	1	12.63	<0.001
TF11	11.98	1	11.98	<0.001
TF1 × TF2	6.98	1	6.98	0.008
SITE × TF2	113.72	4	28.43	<0.001
SITE × TF3	131.88	4	32.97	<0.001
SITE × TF5	19.65	4	4.91	<0.001
SITE × TF7	31.11	4	7.78	<0.001
SITE × TF12	22.49	4	5.62	<0.001

**Figure 3:**
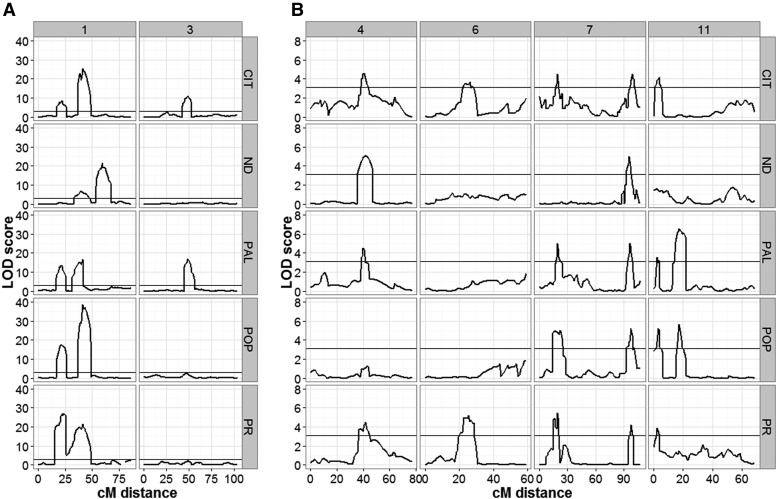
TF QTL profiles across the five experimental sites. (A) LOD profile for QTL detected on chromosomes 1 and 3 at all five sites, (B) QTL detected on chromosomes 4, 6, 7, and 11 at all five sites. Analyses were performed with the WinQTL Cartographer software using the CIM method. The black horizontal line indicates the LOD threshold value for detecting significant QTL peaks. The numbers in the top gray panel represent the chromosome number, while the sites are indicated on the right side gray panel. The bottom *x*-axis represents distance in cM for a given chromosome, while the left *y*-axis represents the LOD score. The five locations include: CIT, ND, PAL, POP, and PR.

Independent QTL analyses using the CIM approach with WinQTL Cartographer 2.5 (Wang *et al.* 2012) detected the same QTL across all sites (Figure 3) on the same six chromosomes (1, 3, 4, 6, 7, and 11) as were detected via the GenStat software. Under this analysis, the most significant QTL were detected on chromosomes 1 and 3, with LOD scores ranging between 10 and 40. Our analysis also showed that several QTL were site-specific, while others varied in their significance level across sites (Figure 3). These results indicate the presence of significant GEI effects. Furthermore, the QTL detected by CIM were incorporated into a QTL model that was used to scan the linkage map using MIM (Zeng *et al.* 1999). This analysis did confirm the significant additive effects of nine QTL in the model (Table S7 in File S1), but was not able to capture the effect of QTL TF4, TF5, and TF8. MIM results also indicated that TF1 and TF2 on chromosome 1 had epistatic interactions at CIT, PR, and PAL. TF2 and TF3 were detected as major QTL in both GenStat and WinQTL analyses.

### A QTL-based TF model

A mixed-effect additive model (*Materials and Methods*, Equation 3) was constructed based on the multi-site QTL mapping model generated by GenStat v.17, with the inclusion of the epistatic effect (as interaction) detected between TF1 and TF2 through MIM. Residual analysis indicated that the model conformed to the assumption of a mixed-effects linear model. The model allowed the QTL effects to be broken down into QTL main effects and QTL-by-environment effects, allowing the assessment of individual QTL effects across environments. The Wald statistic was used to test the significance of each term (Table 4). These tests confirmed that the effects of TF2, TF3, TF5, TF7, and TF12 significantly varied with the environment, and also indicated a significant interaction between TF1 and TF2 (Table 4). Successively, the mixed-effect model (QTL-site-based model) was employed to estimate the flowering time for each RIL at all five sites using the QTL genotype data. Comparison of the predicted to the observed TF across sites indicated a good fit, as represented by an *r*-square of 0.92, and root mean square error (RMSE) of 2.47 d (Figure S2 in File S1).

### Incorporating environmental information into the QTL model

The QTL-site-based model was modified to partition the site effects into individual environmental components following Malosetti *et al.* (2013). This modification allowed the identification and assessment of the main effect of environmental components as well as their interactions with individual QTL. Light and temperature are known to strongly influence TF; therefore, the following environmental components were selected for modeling: DAY (hr), NIGHT (hr), Srad (MJ m^−2^ d^−1^), Tmin (°), Tavg (°), Tmax (°), D_Tavg (°), and N_Tavg (°).

**Figure 4:**
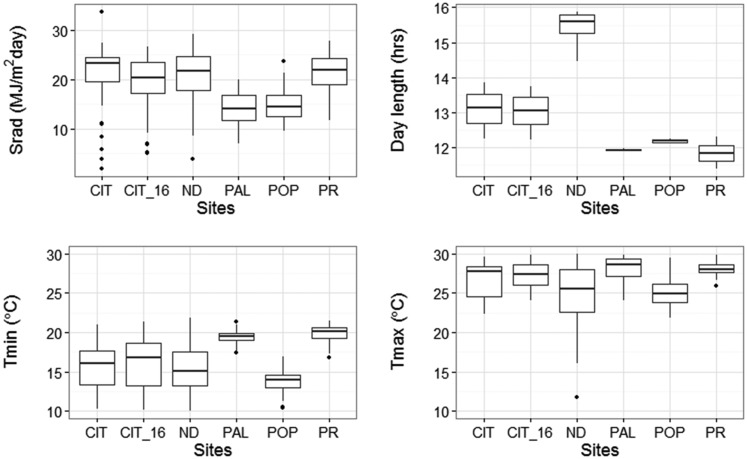
Profile of four environmental variables observed between sowing and flowering time at each of the five experimental sites. Boxplots show spread of average daily solar radiation (top left), average day length (top right), average minimum temperature (bottom left) and average maximum temperature (bottom right). CIT_16 represents the weather data collected during the validation experiment conducted in the year of 2016 at Citra, FL. The five sites include: CIT, ND, PAL, POP, and PR.

**Figure 5:**
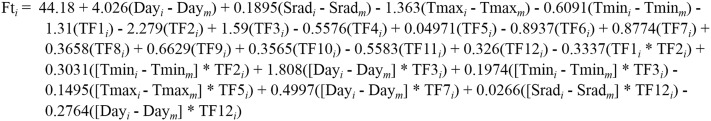
QTL-EC linear model. Ft_i_, flowering time of the *i*th genotype; 44.18, mean TF (day) across the five sites; Day*_i_*, average day length from sowing to flowering observed by the *i*th genotype (hours); Day*_m_*, mean day length across all five sites (12.37 hr); Srad*_i_*, average solar radiation from sowing to flowering observed by the *i*th genotype (Srad, MJ m^–2^ d^–1^); Srad*_m_*, mean solar radiation across all five sites (18.218 MJ m^–2^ d^–1^); Tmin*_i_*, average minimum temperature from sowing to flowering observed by the *i*th genotype (°); Tmin*_m_*, mean minimum temperature across all five sites (16.128°); Tmax*_i_*, average maximum temperature from sowing to flowering observed by *i*th genotype (°); Tmax*_m_*, mean maximum temperature across all five sites (27.458°); TF1*_i_*:TF12*_i_*, alleles at QTL TF1:TF12 in the *i*th genotype (Calima alleles = “+1” and Jamapa allele = “−1”).

An assessment of collinearity between the EC identified four of the least related (ρ < 0.5, Figure S3 in File S1), but biologically meaningful variables for modeling; namely DAY, Srad, Tmin, and Tmax. Variation of the four selected ECs from sowing to the flowering day of the latest RIL across sites is shown in the boxplots of Figure 4. The selected ECs were incorporated into the model by first substituting the “Site” factor in QTL × Site effects with the individual EC explanatory variables, one at a time, and evaluating their significance via the Wald statistic. Subsequently, the main effects of ECs were included in the model to estimate the direct effect of each EC on the flowering time separate from their effect on individual QTL. Wald test results from the QTL-EC model indicated that not only did all ECs had significant main effects, but that they also interacted with specific QTL (Table 5). Tmin had significant interactions with TF2, and TF3, while DAY affected the actions of TF3, TF7, and TF12. Also, TF5 and TF12 interacted with Tmax and Srad, respectively. The QTL-EC model is presented in Figure 5.

**Table 5:** Conditional Wald statistic tests for fixed effects of the TF predictive model incorporating QTL × Environment interactions

Fixed Term	Wald Statistic	Degrees of Freedom (d.f.)	Wald/d.f.	*P*-Value
Day	1011.79	1	1011.79	<0.001
Srad	91.17	1	91.17	<0.001
Tmax	761.86	1	761.86	<0.001
Tmin	273.00	1	273.00	<0.001
TF1	104.95	1	104.95	<0.001
TF2	195.36	1	195.36	<0.001
TF3	68.54	1	68.54	<0.001
TF4	15.06	1	15.06	<0.001
TF5	0.12	1	0.12	0.728
TF6	39.88	1	39.88	<0.001
TF7	41.02	1	41.02	<0.001
TF8	11.32	1	11.32	<0.001
TF9	40.46	1	40.46	<0.001
TF10	10.74	1	10.74	0.001
TF11	11.59	1	11.59	<0.001
TF12	3.06	1	3.06	0.08
TF1 × TF2	6.64	1	6.64	0.01
Tmin × TF2	117.08	1	117.08	<0.001
Day × TF3	224.12	1	224.12	<0.001
Tmin × TF3	31.58	1	31.58	<0.001
Tmax × TF5	14.31	1	14.31	<0.001
Day × TF7	31.63	1	31.63	<0.001
Srad × TF12	7.79	1	7.79	0.005
Day × TF12	9.53	1	9.53	0.002

The model has 12 QTL (TF1–TF12) and four environmental factors averaged over the time span between planting and first anthesis of the last genotype at each site. Tmin (avg. minimum temperature, °), Tmax (avg. maximum temperature, °), Day (avg. day length, hr), and Srad (avg. solar radiation, MJ m^−2^ d^−1^).

Parameters estimated for the QTL-EC model (Figure 5) indicated that a 1-hr increase in day length during vegetative development delayed the population mean TF (across sites) by, on average, 4.03 ± 0.13 d, while a unit (°) increase in Tmin and Tmax reduced mean TF by 0.61 ± 0.04 and 1.36 ± 0.05 d, respectively (Table S8 in File S1). The QTL-EC model further estimated the main effect of each QTL (Table S8 in File S1). For example, TF2 was estimated to reduce the mean flowering time by 2.28 ± 0.16 d when homozygous for the Calima allele, but it has the opposite effect when homozygous for the Jamapa allele. The model also captured QTL-by-environment interaction effects. For example, modeling TF2 with Tmin as covariable showed a significant change in the TF2 effect with the change in the minimum temperature (Table 5). Of note, the model predictability is expected to be reliable only within the environmental ranges observed across environments during the experimental periods.

**Figure 6:**
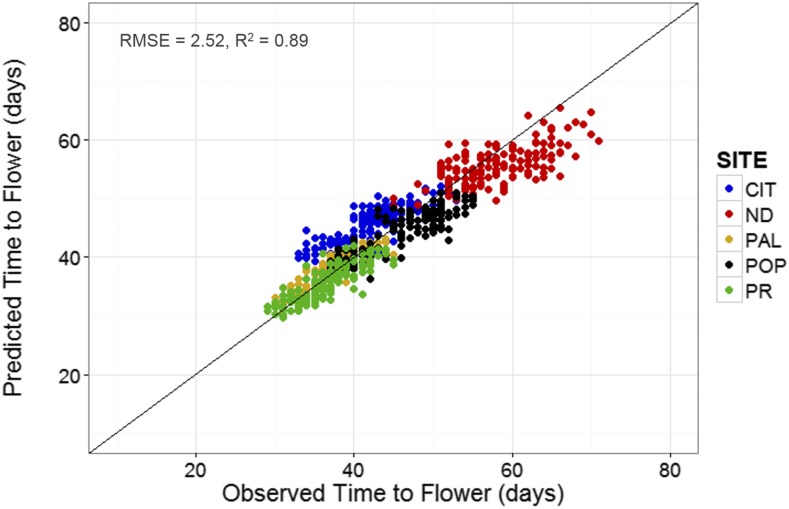
Predicted *vs.* observed values of TF at the five experimental sites. The predicted values were obtained using the QTL-EC model (QTL + environmental covariates model). Dots represent recombinant inbred lines. The five sites include: CIT, ND, PAL, POP, and PR.

**Figure 7:**
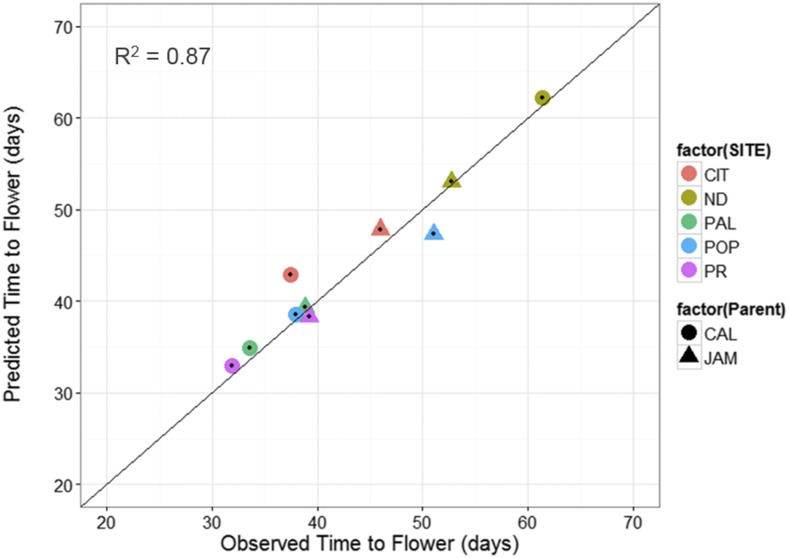
QTL-EC model evaluation by predicting TF for the parental genotypes grown at the five sites. The predicted values were obtained using the QTL-EC model (QTL + environmental covariates model). The parental genotypes Calima (CAL, solid circles) and Jamapa (JAM, solid triangle) were not included in the model parameter estimation process. The five sites include: CIT, ND, PAL, POP, and PR.

**Figure 8:**
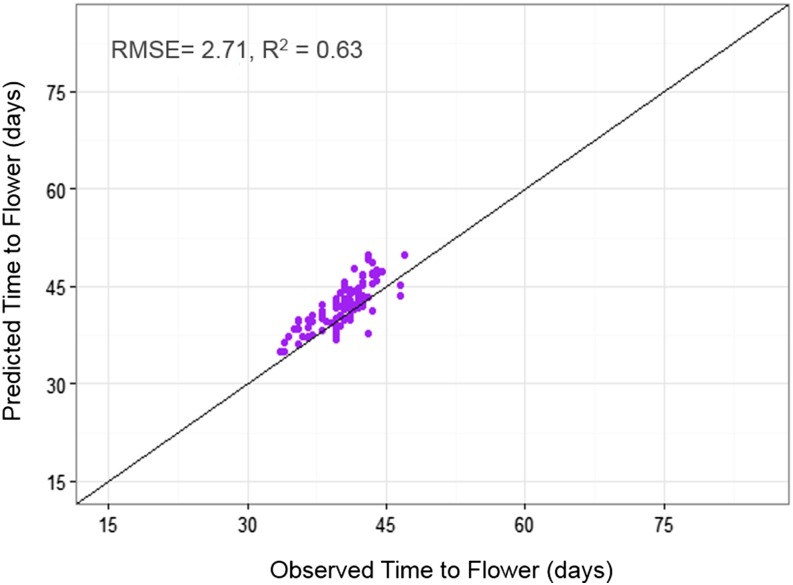
QTL-EC model evaluation by predicting TF for 100 RILs grown in 2016 at Citra, FL (CIT_16). The predicted values were obtained using the QTL-EC model (QTL + environmental covariates model). The parameter estimation process did not included Citra, FL 2016 (CIT_16) data.

Lastly, the QTL-EC model was used to re-estimate TF for all RI lines across sites. A comparison of the predicted with the observed TF across sites showed an adjusted *r*-square value of 0.89 with a RMSE of 2.52 d (Figure 6 and Table 6). The QTL-EC model’s performance was evaluated in three different ways. First, by predicting the TF of both parental lines, which were purposely left out during parameter estimation. The predicted parental TFs at all five sites yielded an adjusted *r*-square value of 0.87 (Figure 7). Second, the estimation of TF for 100 lines of the RIL population grown again at Citra, FL (CIT_16) in the year 2016 (Figure S5 in File S1) indicated that the QTL-EC model was able to estimate TF with an adjusted *r*-squared value of 0.63, and RMSE of 2.71 d (Figure 8). These results indicate that the model is capable of predicting TF across years at locations used in training the model. Lastly, the model performance was tested by estimating parameters using data from only four primary sites, and then predicting TF for the genotypes grown at the fifth site (a cross-validation approach). However, this cross-validation reported varying model performance. The model predicted flowering time at PR and PAL with adjusted *r*-squared values of 0.67 and 0.76, and RMSEs of 2.8 and 2.07 d, respectively (Figure S4 and Table S9 in File S1). In contrast, the model predicted TF poorly in ND when the parameters were estimated using data from the other four primary sites, yielding an adjusted *r*-squared value of 0.42, and a RMSE of 25.83 d (Figure S4 and Table S9 in File S1).

**Table 6:** One-site-out evaluation of the TF predictive model (QTL+ environmental covariate based model) performance for the RI population grown at the five sites: PR, ND, POP, CIT, and PAL

	CIT	ND	PAL	POP	PR	Overall
RMSE (d)	2.45	3.93	1.42	2.16	1.87	2.47
Adjusted *r*^2^	0.73	0.48	0.79	0.81	0.73	0.92

## Discussion

The frequency distributions of TF at each of the five sites clearly indicated that TF is a polygenic trait, and that site-to-site changes in the distribution patterns were indicative of strong GEI. This phenomenon was best demonstrated in the reversal in TF of the parents in ND, and the change to a bimodal pattern observed in POP. The latter pattern is suggestive of a gene with a predominant effect under the conditions of the POP site, which had the lowest temperature and short days. In addition, the transgressive behavior of some RILs suggested that both parents possess some alleles that shorten, and others that delay, TF. The observed variation for TF was largely due to genetic effects, as evidenced by the broad-sense heritability estimate of 0.78. Such high heritability is not uncommon for this trait, as it has also been reported in other species like maize (Buckler *et al.* 2009), tomato (Mohamed *et al.* 2012), and rice (Seyoum *et al.* 2012). The high heritability estimate for TF indicated a high correlation between the phenotypic and genetic values, which increased the power of QTL detection.

The genetic complexity of the TF trait was highlighted by the detection of a total of 12 TF QTL in the RIL population. The importance of chromosome 1 in the control of TF has been reported previously (Gu *et al.* 1998; Pérez-Vega *et al.* 2010), and was underscored by the detection of four associated QTL, three of which have large effects, and were identified with LOD values between 10 and 40. The preliminary observation of GEI was supported by our analysis with the mixed-effects statistical model, which revealed that five QTL interacted with the environment, whereas the remaining seven were detected as environmentally insensitive. These environmental interactions indicate that the common bean, like many other species, including *Arabidopsis* (Mouradov *et al.* 2002; Simpson and Dean 2002; Moon *et al.* 2005), uses environmental cues to switch from the vegetative to the reproductive phase. Thus, identification of these QTL would be of great breeding importance in the development of cultivars adapted to targeted environments.

TF2 significant interactions with the average Tmin could explain, to a great extent, the bimodal pattern observed in POP, the high altitude equatorial site with the lowest temperatures (as well as short day length). The chromosome region associated with TF2 encompasses the *FIN* locus (Repinski *et al.* 2012), which controls growth habit in the common bean and explains why the early flowering mode predominantly included determinate RILs, while the late flowering mode predominantly included indeterminate RILs. It is possible that TF2 (43.5–46.0 Mbp, Chr1) and *FIN* (45.5 Mbp, Chr1, Phvul.001G189200) are tightly linked loci. However, a number of observations suggest that these may be the same gene, and the behavior of TF2 represents the pleiotropic effects of *FIN*, which was found to be a homolog of the *Arabidopsis TFL1* gene (Repinski *et al.* 2012). The *Arabidopsis TFL1* gene controls growth habit by repressing floral development in the shoot apical meristem (Shannon and Meeks-Wagner 1991), and it also acts as a temperature sensor delaying flowering at low temperatures (Strasser *et al.* 2009; Kim *et al.* 2013). Mutant alleles of this gene cause the development of a terminal inflorescence, and fail to delay flowering at low temperatures. A similar temperature effect has also been reported for the homolog FvTFL1 in transgenic strawberry (Rantanen *et al.* 2015). The *FIN* allele responsible for determinacy in beans is known to have a deletion (Repinski *et al.* 2012), and could also have a similar pleiotropic function in common bean controlling a temperature dependent flowering pathway. In this way, *FIN* could explain the bimodal distribution of flowering associated with growth habit detected in POP site. These observations are in agreement with those of Wallace *et al.* (1991) and Kornegay *et al.* (1993), who reported that flowering of indeterminate genotypes is significantly delayed at low temperatures. Taken together, these observations suggest that *FIN* could be considered as a strong candidate for TF2.

At the upper range of the temperature scale, TF5 displayed significant interactions with Tmax. In fact, TF2 and TF5 could explain the U-shape TF response of beans reported by Wallace *et al.* (1991). Thus, the rate at which TF is increased could be controlled by TF2 and TF5 by reduction and increases in Tmin and Tmax, respectively.

TF3 was shown to play a major role in conditioning sensitivity to day length, causing a minimal effect on flowering time at PR and PAL (short day sites), but explaining a major fraction of the variation in ND (long day site). The chromosome segment associated with TF3 appears to coincide with the region where the previously recognized dominant photoperiod sensitive gene “*Ppd*” was mapped (Gu *et al.* 1998), raising the possibility that TF3 and *Ppd* could be the same locus. The absence of sites with clear factorial temperature and photoperiod combinations kept us from assessing the full range of effect of the interactions between these variables on TF as described in the literature (White and Laing 1989; Wallace *et al.* 1991; Kornegay *et al.* 1993; White *et al.* 1996).

An intriguing result was the significant effect of solar radiation on TF, and the significant interaction between TF12 and solar radiation. In fact, the amount of solar radiation is known to affect the timing of flowering (Adams *et al.* 1997). However, not much is known about the exact mechanism, but some observations suggest the control of Red:Far-red (R:FR) ratios might be involved. Although cloud coverage is considered by many to have a neutral effect, measurements indicate that cloud coverage reduces downwelling solar irradiance, but it also changes the spectrum, particularly at the red end of the PAR (Bartlett *et al.* 1998). Hence, differential sensitivity to perceived R:FR ratios caused by cloud coverage may explain interactions between TF12 and both solar radiation and photoperiod as observed in other systems (Kurepin *et al.* 2007; Martínez-García *et al.* 2014).

The mixed-effects model helped us assess the contribution of genetic, environmental, and G × E components to the observed variation in TF. The QTL-EC model proved to be an effective predictor of TF, requiring the TF-QTL genotype and environmental information as the only inputs. In fact, the QTL-EC model predicted very well all transgressive TF phenotypes, which resulted from the fact that Jamapa alleles at TF1, 2, 4, 6, and 11 delayed TF, while the opposite was true for TF3, 7, 8, 9, 10, and 12. This observation highlights the richness of the two common bean gene pools for alleles that control the timing of the transition from the vegetative to the reproductive phase, and suggests that a greater amount of variation probably exists in these gene pools. The QTL-EC modeling approach could be very useful in plant breeding programs, and it can contribute to facilitate development of ideotypes for specific environments. The model will also be useful for germplasm conversion programs where the objective is to move traits between germplasm adapted to tropical and temperate environments.

Although the evaluation with both the parental and additional 2016 data sets showed the robustness of the QTL-EC model at all the environments under study, the predictive accuracy of this model, or models in general, is restricted to the range of experimental conditions and experimental data collected (Malosetti *et al.* 2013). For this reason, this model should be considered as a starting point for the construction of a more complete model, which could be accomplished by the inclusion of a wider range of environments and a diversity panel that represents the variation in the germplasm bank. Increasing diversity in general is bound to alter the model’s center point and the slope of environmental sensitivities.

The variation not explained by the model indicates that all the components of variation may not have been identified. For instance, it is possible that several QTL with very small effects were not detected due to the relatively small size of the population. In addition, the reduction in predictability resulting from the shift from the QTL-Site to the QTL-EC model suggests that perhaps one or more environmental variables at the experimental sites were not taken into account. This issue can be appreciated more clearly by the reduced efficiency of the model when one of the sites is left out of the calibration, particularly the ND site (Figure S4 in File S1). The most likely explanation for the largest deviations from the model observed in ND is the absence of a site with long days and temperatures as low as those from POP. The absence of such a site prevented us from capturing the nonlinear effect on TF caused by interactions between temperature and photoperiod reported by Wallace *et al.* (1991). Inclusion of this type of interaction, and others that may manifest more markedly at extreme environmental conditions, will require the use of nonlinear models.

Further studies are being conducted to validate and map with greater precision the TF QTL detected in this study. These efforts will facilitate the identification of each QTL. The current data strongly suggest the TF2 may correspond to PvTF1, and TF3 may be correspond to a photoperiod responsive gene, like one of the phytochromes found in that region. However, TF1 was detected within a peak of ∼10 cM, but the centimorgan to Mega base pair relationship in this region of chromosome 1 indicates that this segment stretches over 20–30 Mbp (Bhakta *et al.* 2015). Thus, at this point, it would be premature to speculate about the identities of the other QTL. We must also point out that this study was restricted to a single biparental cross, and used only five environmental sites. Thus, it is possible that alternative alleles of the QTL reported here, or additional loci involved in the control of TF, may be present in the common bean germplasm. For instance, the survey of White and Laing (1989) showed that Calima belongs to one (Class 5) of eight photoperiod response groups. Furthermore, additional combinations of environmental factors should be tested to obtain a more complete assessment of the environmental effects as indicated above.

In summary, this study identified 12 significant quantitative loci controlling TF in the common bean. Development of a QTL-based environmental linear mixed effect model allowed identification of several QTL that interacted with specific environmental factors like temperature, photoperiod and solar radiation. The mixed-effect model predicted TF with good accuracy, and allowed us to improve our understanding of the genetic and physiological mechanisms that control flowering in the common bean. These results suggest that *in silico* testing of the performance of different QTL allele combinations under specific environmental conditions could help breeders identify and design adapted elite varieties, thereby saving time and resources.

## Supplementary Material

Supplemental material is available online at www.g3journal.org/lookup/suppl/doi:10.1534/g3.117.300229/-/DC1.
