# Supplementary material for: A Predictive Model for Time-to-Flowering in the Common Bean Based on QTL and Environmental Variables
Source: G3 (Bethesda). 2017 Oct 12;7(12):3901–12. doi: 10.1534/g3.117.300229 (PMC5714487; doi:10.1534/g3.117.300229)
Supplement: Supplementary file 1 [file 3901FileS1.docx]

# SUPPLEMENTARY INFORMATION

# Figures


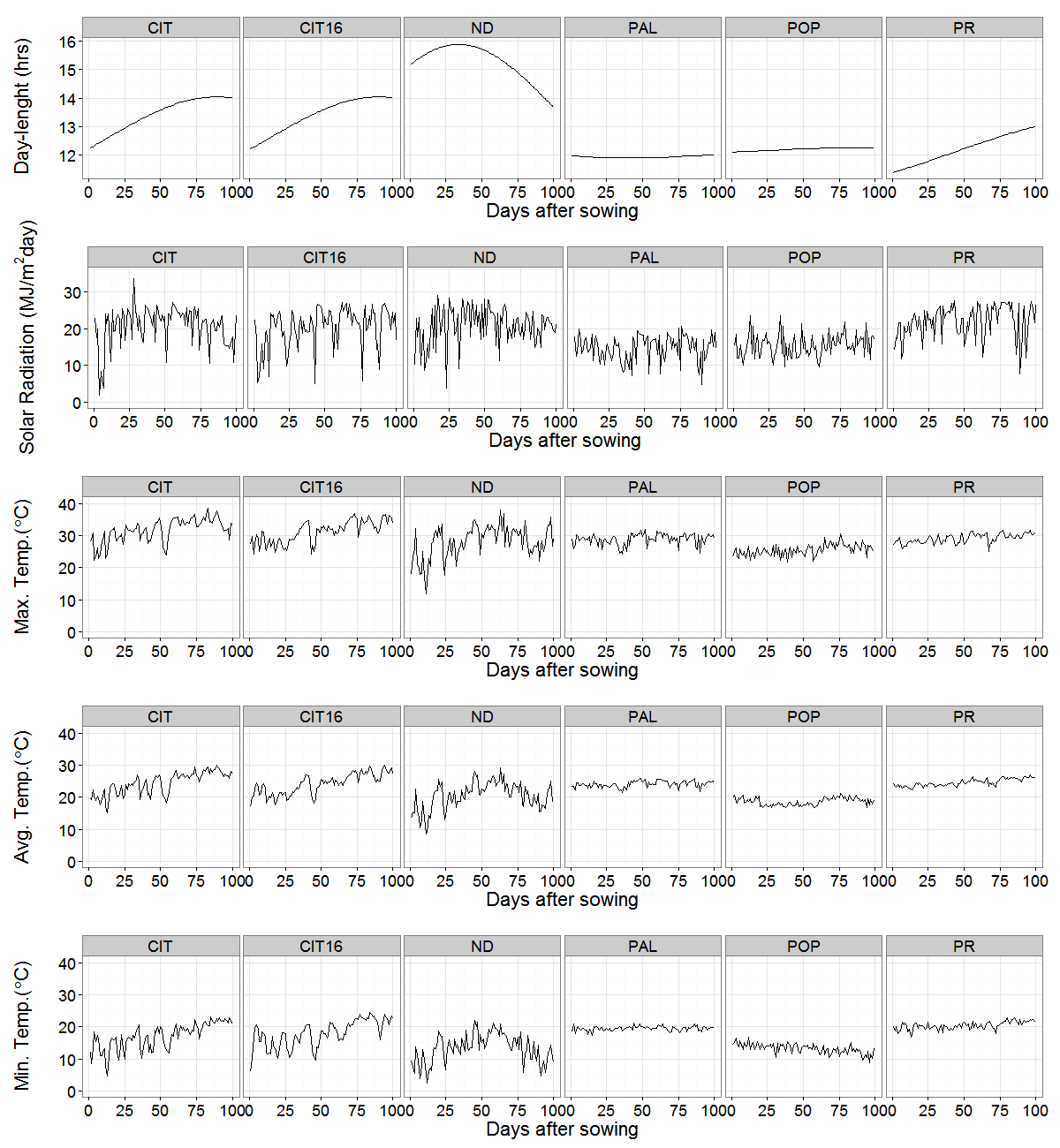


**Figure S1. Profiles of climatic variables observed during the first 100 days after sowing at five primary locations and one validation location.**

Primary experimental locations include Citra, FL (CIT), North Dakota (ND), Palmira, (PAL), Popayan, (POP) and Puerto Rico (PR). Validation experimental location include Citra, FL (CIT_16).

**
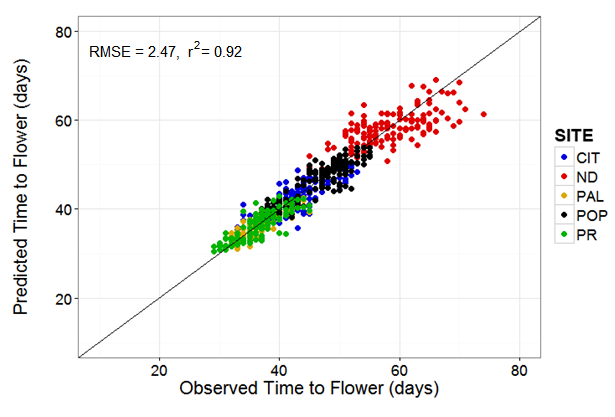
**

**Figure S2. Predicted vs observed values of time to flower for RI individuals grown at five locations.**

The predicted values were obtained using the QTL-based mixed-effect model. Experimental locations include Citra, FL (CIT), North Dakota (ND), Palmira, (PAL), Popayan, (POP) and Puerto Rico (PR).

**
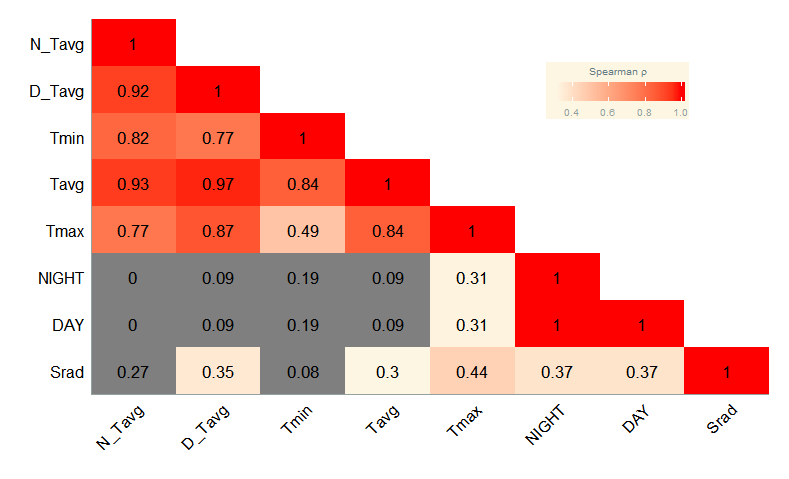
**

**Figure S3. Correlations matrix of averaged environmental variables observed between the sowing and flowering dates.**

DAY and NIGHT indicates daylight and night time durations in hours; Srad indicates average solar radiation (MJ m^-2^ d^-1^); Tmin, Tavg, Tmax, N_Tavg, and D_Tavg as minimum, average, maximum, average day time and average night time temperatures in ˚C.

**
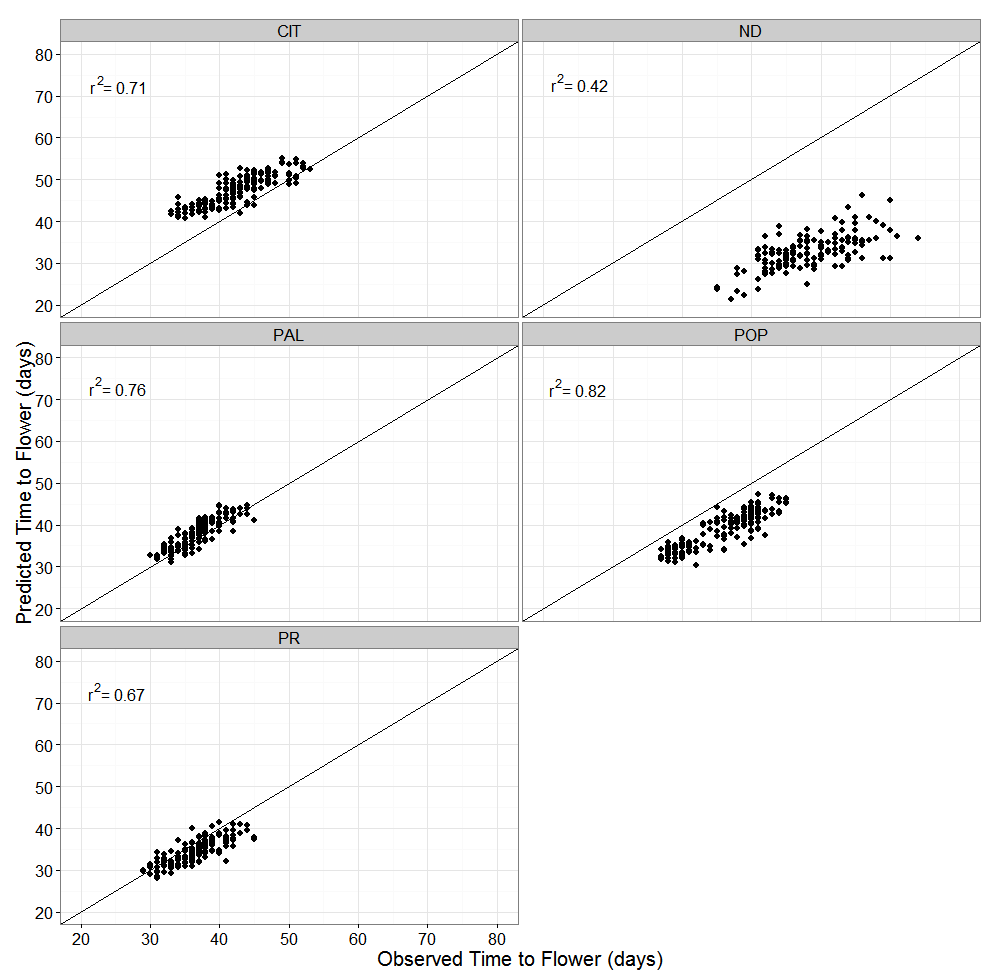
**

**Figure S4. Evaluation of the QTL-EC model performance by prediciting time-to-flower at one site using parameters estimated with the combined other four site data.** Scatter-plot comparing predicted values to the observed values at each of the five sites: Citra, FL, USA (CIT), North Dakota, USA (ND), Palmira, Colombia (PAL), Popayan, Colombia (POP), and Puerto Rico (PR).


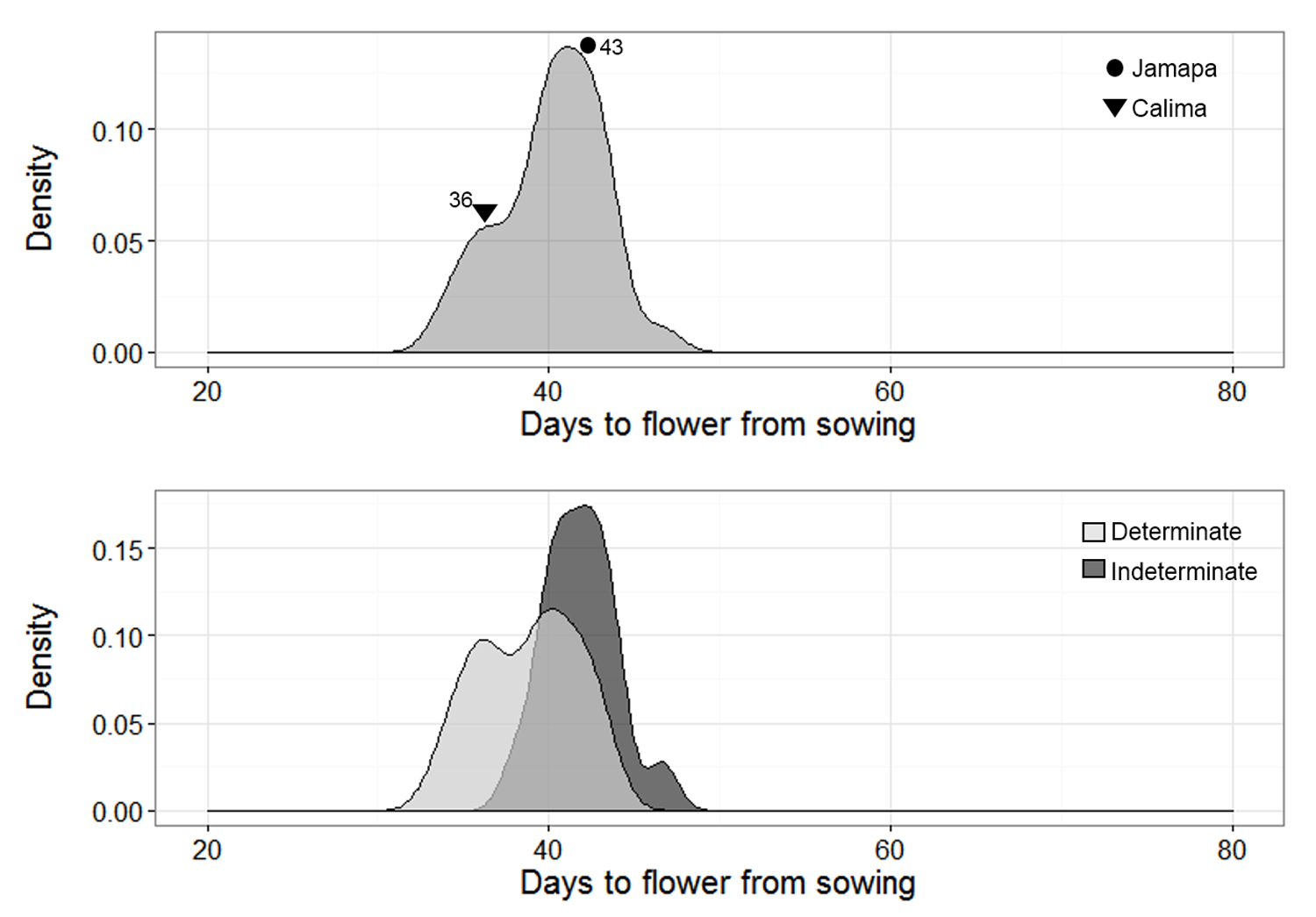
 **Figure S5. Density plots of days to first flower observed at Citra, FL in 2016 (CIT_16).**

Top- Distribution of days to flower where the solid black triangle represent the number of days that Calima took to first flower, while the solid black circle represent the number of days for Jamapa. Bottom- Distribution of days to flower based on growth habit: The light grey area indicates TF distribution of determinate RI lines, while dark grey area represent indeterminate RI lines.

# Tables

**Table S1. Basic field trial information for experimental locations utilized for phenotyping the recombinant inbred lines for time to flower. Five primary locations include Citra, Florida, USA (CIT), Prosper, North Dakota, USA (ND), Isabela, Puerto Rico (PR), Palmira, Colombia (PAL), and Popayan, Colombia (POP). Additional year data were collected at the Citra location in Florida (CIT_16) for the model evaluation purpose.**

| **Location** | **Type** | **Sowing Date** | **Irrigation** | **Plot Density (plants/m^2^)** | **Row spacing**  **(cm)** | **No. of RIL** |
| --- | --- | --- | --- | --- | --- | --- |
| CIT | Primary | 24-Mar, 2011 | Central Pivot | 4.3 | 90 | 168 |
| ND | Primary | 10-May, 2012 | Rain fed | 3.3 | 75 | 154 |
| PAL | Primary | 11-Nov, 2011 | Furrow | 3 | 120 | 171 |
| POP | Primary | 23-Mar, 2012 | Rain fed | 4.3 | 90 | 171 |
| PR | Primary | 06-Feb, 2012 | Drip irrigation | 3.9 | 100 | 161 |
| CIT_16 | Validation | 23-Mar, 2016 | Central Pivot | 4.3 | 90 | 100 |

**Table S2. Statistical analysis of variance based on time to first flower scores (days) in RI population grown at the five primary sites: Florida (CIT), North Dakota (ND), Puerto Rico (PR), Palmira (PAL), and Popayan (POP).**

| **Source of Variance** | **Df** | **Sum Sq** | **Mean Sq** | **F value** | **P Value** |
| --- | --- | --- | --- | --- | --- |
| SITE | 4 | 48880 | 12220.1 | 1230.07 | <0.001 |
| RIL | 186 | 10668 | 57.4 | 5.77 | <0.001 |
| Residuals | 630 | 6259 | 9.9 |  |  |

**Table S3. Descriptive statistics for time to flower observed in the recombinant inbred line population grown at the five sites: Florida (CIT), North Dakota (ND), Puerto Rico (PR), Palmira (PAL), and Popayan (POP).**

|  | **Time to Flower (days)** | | | | |  |
| --- | --- | --- | --- | --- | --- | --- |
| **Site** | **Mean** | **Median** | **Std. Dev.** | **Min** | **Max** | **Bonferroni's grouping** |
| CIT | 42.5 | 42.5 | 4.7 | 33 | 53 | A |
| ND | 57.8 | 57.0 | 5.5 | 45 | 71 | B |
| PAL | 36.7 | 37.0 | 3.1 | 30 | 45 | C |
| PR | 36.4 | 37.0 | 3.6 | 29 | 45 | C |
| POP | 46.0 | 47.0 | 5.2 | 37 | 55 | D |

**Table S4.** **Broad-sense heritabilities (*H_s_^2^*) for time to flower (TF) estimated using site-specific mixed-effect models. Estimations were made for the RI population grown at the five sites: Isabela, Puerto Rico (PR), Prosper, North Dakota (ND), Popayan (POP), Citra, FL (CIT), and Palmira (PAL).**

|  | **Site-level variance components** | | | | |
| --- | --- | --- | --- | --- | --- |
| **Variance Component** | **PR** | **ND** | **POP** | **CIT** | **PAL** |
| Rep x Row | 0.166 | 1.177 | 0.000 | 0.189 | 0.000 |
| Rep x Col | 0.595 | 0.500 | 0.516 | 0.287 | 0.178 |
| RIL | 2.926 | 14.362 | 2.649 | 3.533 | 0.898 |
| Error | 11.877 | 35.628 | 25.881 | 20.045 | 9.040 |
| Total | 3.687 | 16.039 | 3.166 | 4.008 | 1.076 |
| *H_s_^2^* | 0.763 | 0.690 | 0.891 | 0.833 | 0.894 |

**Table S5. Variance-covariance model selection for phenotypic data (time to flower) prior to running single interval mapping in GenStat 17.**

| **Model** | **AIC** | **Deviance** | **No. of Parameters** |
| --- | --- | --- | --- |
| Unstructured | 2505 | 2475 | 15 |
| First order factor-analytic | 2515 | 2495 | 10 |
| Heterogeneous compound symmetry | 2640 | 2628 | 6 |
| Compound symmetry | 2983 | 2979 | 2 |
| Diagonal | 3218 | 3208 | 5 |

**Table S6. Time-to-flower QTL detected using a mixed-effect model in GenStat 17. Multi-site QTL mapping was carried for the RI family grown at the five sites: Isabela, Puerto Rico (PR), Prosper, North Dakota, USA (ND), Popayan, Colombia (POP), Citra, FL, USA (CIT), and Palmira, Colombia (PAL).**

| **QTL** | **Site** | **High value allele*** | **Calima allele effect (days)** | **Std. Error** | **% Variation Explained** | **p-value** |
| --- | --- | --- | --- | --- | --- | --- |
| TF1 | CIT | Jamapa | -1.37 | 0.13 | 8.40 | <0.001 |
|  | ND | Jamapa | -1.37 | 0.13 | 6.20 | <0.001 |
|  | PAL | Jamapa | -1.37 | 0.13 | 19.60 | <0.001 |
|  | POP | Jamapa | -1.37 | 0.13 | 7.00 | <0.001 |
|  | PR | Jamapa | -1.37 | 0.13 | 14.40 | <0.001 |
| TF2 | CIT | Jamapa | -3.13 | 0.25 | 35.40 | <0.001 |
|  | ND | Jamapa | -2.62 | 0.45 | 15.40 | <0.001 |
|  | PAL | Jamapa | -1.37 | 0.16 | 19.70 | <0.001 |
|  | POP | Jamapa | -3.16 | 0.23 | 37.20 | <0.001 |
|  | PR | Jamapa | -1.85 | 0.20 | 26.30 | <0.001 |
| TF3 | CIT | Calima | 1.28 | 0.27 | 7.30 | <0.001 |
|  | ND | Calima | 5.37 | 0.47 | 39.20 | <0.001 |
|  | PAL | Calima | 0.65 | 0.18 | 4.50 | <0.001 |
|  | POP | Calima | 0.16 | 0.25 | 0.10 | 0.519 |
|  | PR | Calima | 0.39 | 0.23 | 1.20 | 0.084 |
| TF4 | CIT | Jamapa | -0.60 | 0.15 | 1.60 | <0.001 |
|  | ND | Jamapa | -0.60 | 0.15 | 1.20 | <0.001 |
|  | PAL | Jamapa | -0.60 | 0.15 | 3.80 | <0.001 |
|  | POP | Jamapa | -0.60 | 0.15 | 1.40 | <0.001 |
|  | PR | Jamapa | -0.60 | 0.15 | 2.80 | <0.001 |
| TF5 | CIT | Jamapa | -0.53 | 0.22 | 1.20 | 0.019 |
|  | ND | Jamapa | -0.01 | 0.35 | 0.00 | 0.975 |
|  | PAL | Jamapa | -0.08 | 0.16 | 0.10 | 0.620 |
|  | POP | Calima | 0.35 | 0.21 | 0.50 | 0.094 |
|  | PR | Calima | 0.04 | 0.19 | 0.00 | 0.836 |
| TF6 | CIT | Jamapa | -0.92 | 0.15 | 3.80 | <0.001 |
|  | ND | Jamapa | -0.92 | 0.15 | 2.80 | <0.001 |
|  | PAL | Jamapa | -0.92 | 0.15 | 8.90 | <0.001 |
|  | POP | Jamapa | -0.92 | 0.15 | 3.20 | <0.001 |
|  | PR | Jamapa | -0.92 | 0.15 | 6.50 | <0.001 |
| TF7 | CIT | Calima | 0.98 | 0.20 | 4.30 | <0.001 |
|  | ND | Calima | 2.00 | 0.34 | 9.60 | <0.001 |
|  | PAL | Calima | 0.39 | 0.12 | 1.60 | 0.001 |
|  | POP | Calima | 0.42 | 0.19 | 0.70 | 0.023 |
|  | PR | Calima | 0.50 | 0.16 | 1.90 | 0.002 |

**Table S6. Continue**

| **QTL** | **Site** | **High value allele*** | **Calima allele effect (days)** | **Std. Error** | **% Variation Explained** | **P-value** |
| --- | --- | --- | --- | --- | --- | --- |
| TF8 | CIT | Calima | 0.39 | 0.11 | 0.70 | 0.001 |
|  | ND | Calima | 0.39 | 0.11 | 0.50 | 0.001 |
|  | PAL | Calima | 0.39 | 0.11 | 1.60 | 0.001 |
|  | POP | Calima | 0.39 | 0.11 | 0.60 | 0.001 |
|  | PR | Calima | 0.39 | 0.11 | 1.20 | 0.001 |
| TF10 | CIT | Calima | 0.43 | 0.11 | 0.80 | <0.001 |
|  | ND | Calima | 0.43 | 0.11 | 0.60 | <0.001 |
|  | PAL | Calima | 0.43 | 0.11 | 2.00 | <0.001 |
|  | POP | Calima | 0.43 | 0.11 | 0.70 | <0.001 |
|  | PR | Calima | 0.43 | 0.11 | 1.40 | <0.001 |
| TF9 | CIT | Calima | 0.66 | 0.11 | 2.00 | <0.001 |
|  | ND | Calima | 0.66 | 0.11 | 1.50 | <0.001 |
|  | PAL | Calima | 0.66 | 0.11 | 4.60 | <0.001 |
|  | POP | Calima | 0.66 | 0.11 | 1.60 | <0.001 |
|  | PR | Calima | 0.66 | 0.11 | 3.40 | <0.001 |
| TF11 | CIT | Jamapa | -0.64 | 0.17 | 1.80 | <0.001 |
|  | ND | Jamapa | -0.64 | 0.17 | 1.30 | <0.001 |
|  | PAL | Jamapa | -0.64 | 0.17 | 4.30 | <0.001 |
|  | POP | Jamapa | -0.64 | 0.17 | 1.50 | <0.001 |
|  | PR | Jamapa | -0.64 | 0.17 | 3.10 | <0.001 |
| TF12 | CIT | Calima | 0.43 | 0.23 | 0.80 | 0.063 |
|  | ND | Calima | 1.46 | 0.36 | 7.10 | <0.001 |
|  | PAL | Calima | 0.06 | 0.17 | 0.00 | 0.718 |
|  | POP | Jamapa | -0.02 | 0.22 | 0.00 | 0.946 |
|  | PR | Calima | 0.35 | 0.20 | 0.90 | 0.084 |

*High allele value indicate the parental allele that delays flowering time.

**Table S7. Time to flower QTL detected using multiple-interval mapping (MIM). Single site QTL mapping was carried for the RI family grown at the five sites: Isabela, Puerto Rico (PR), Prosper, North Dakota, USA (ND), Popayan, Colombia (POP), Citra, FL, USA (CIT), and Palmira, Colombia (PAL). MIM detected QTL with additive effects (A) and QTL- by-QTL interaction (E). Certain QTLs were not detected at some sites (-).**

| **QTL**  **(GenStat QTL)** | **Linkage Group** | **Position (cM)** | **Site** | **LOD** | **Calima allele Effect (days)** | **% Variation Explained** | **Type** |
| --- | --- | --- | --- | --- | --- | --- | --- |
| 1 (TF1) | 1 | 22.11 | CIT | 8.29 | -1.45 | 18.00 | A |
|  |  |  | ND | 3.77 | -1.32 | 2.50 | A |
|  |  |  | PAL | 16.66 | -1.31 | 27.20 | A |
|  |  |  | POP | 17.11 | -2.66 | 15.20 | A |
|  |  |  | PR | 13.49 | -1.35 | 23.70 | A |
| 2 (TF2) | 1 | 42.11 | CIT | 20.55 | -2.53 | 36.40 | A |
|  |  |  | ND | 7.25 | -2.27 | 2.70 | A |
|  |  |  | PAL | 13.33 | -1.16 | 25.20 | A |
|  |  |  | POP | 16.24 | -3.65 | 57.00 | A |
|  |  |  | PR | 15.59 | -1.49 | 28.50 | A |
| 3 (TF3) | 1 | 58.81 | CIT | - | - | - | A |
|  |  |  | ND | 21.66 | 4.63 | 32.80 | A |
|  |  |  | PAL | - | - | - | A |
|  |  |  | POP | - | - | - | A |
|  |  |  | PR | - | - | - | A |
| 4 (TF6) | 3 | 45.21 | CIT | 10.67 | -1.43 | 7.50 | A |
|  |  |  | PAL | 16.04 | -1.40 | 11.80 | A |
|  |  |  | PR | 3.02 | -0.49 | 2.10 | A |
|  |  |  | POP | 11.43 | -1.40 | 3.00 | A |
|  |  |  | ND | 4.80 | -1.39 | 4.70 | A |
| 5 (TF7) | 4 | 36.61 | CIT | 3.25 | 0.78 | 4.10 | A |
|  |  |  | ND | 9.61 | 2.04 | 9.10 | A |
|  |  |  | PAL | - | - | - | A |
|  |  |  | POP | - | - | - | A |
|  |  |  | PR | - | - | - | A |
| 6 (TF9) | 7 | 12.71 | CIT | - | - | - | A |
|  |  |  | ND | - | - | - | A |
|  |  |  | PAL | - | - | - | A |
|  |  |  | POP | 5.39 | 0.65 | 2.10 | A |
|  |  |  | PR | 2.38 | 0.57 | 4.30 | A |
| 7 (TF10) | 7 | 103.41 | CIT | 1.86 | 0.60 | 1.00 | A |
|  |  |  | ND | - | - | - | A |
|  |  |  | PAL | - | - | - | A |
|  |  |  | POP | 3.54 | 0.56 | 0.50 | A |
|  |  |  | PR | - | - | - | A |

**Table S7. Continue**

| **QTL**  **(GenStat QTL)** | **Linkage Group** | **Position (cM)** | **Site** | **LOD** | **Calima allele Effect (days)** | **% Variation Explained** | **Type** |
| --- | --- | --- | --- | --- | --- | --- | --- |
| 8 (TF11) | 11 | 2.20 | CIT | - | - | - | A |
|  |  |  | ND | - | - | - | A |
|  |  |  | PAL | - | - | - | A |
|  |  |  | POP | 5.14 | -0.61 | 2.60 | A |
|  |  |  | PR | 4.56 | -0.60 | 3.20 | A |
| 9 (TF12) | 11 | 19.31 | CIT | - | - | - | A |
|  |  |  | ND | - | - | - | A |
|  |  |  | PAL | 4.14 | -0.48 | 2.60 | A |
|  |  |  | POP | - | - | - | A |
|  |  |  | PR | - | - | - | A |
| 1 x 2 | - | - | CIT | 2.80 | 0.70 | 2.30 | E |
|  |  |  | ND | - | - | - | E |
|  |  |  | PAL | - | - | - | E |
|  |  |  | POP | 14.38 | 1.53 | 6.20 | E |
|  |  |  | PR | 2.05 | 0.41 | 1.30 | E |

**Table S8. Main effects of the QTL and the environmental covariates along with their interaction effects as estimated by the time-to-flower predictive QTL-EC model (QTL + environmental covariates based model). QTL effects (TF#) are represented according to the Calima allele.**

| **Model terms** | **Effects (days)** | **Standard Error** |
| --- | --- | --- |
| Day | 4.03 | 0.13 |
| Srad | 0.19 | 0.02 |
| Tmax | -1.36 | 0.05 |
| Tmin | -0.61 | 0.04 |
| TF1 | -1.31 | 0.13 |
| TF2 | -2.28 | 0.16 |
| TF3 | 1.59 | 0.19 |
| TF4 | -0.56 | 0.14 |
| TF5 | 0.05 | 0.14 |
| TF6 | -0.89 | 0.14 |
| TF7 | 0.88 | 0.14 |
| TF8 | 0.37 | 0.11 |
| TF9 | 0.66 | 0.10 |
| TF10 | 0.36 | 0.11 |
| TF11 | -0.56 | 0.16 |
| TF12 | 0.33 | 0.19 |
| TF1 x TF2 | -0.33 | 0.13 |
| Tmin x TF2 | 0.30 | 0.03 |
| Day x TF3 | 1.81 | 0.12 |
| Tmin x TF3 | 0.20 | 0.04 |
| Tmax x TF5 | -0.15 | 0.04 |
| Day x TF7 | 0.50 | 0.09 |
| Srad x TF12 | 0.03 | 0.02 |
| Day x TF12 | -0.28 | 0.09 |

**Table S9. Performance of QTL-EC model (QTL+ Environmental Covariate based model) at predicting flowering time with a site left out from the parameter estimation process. Performance was tested for data observed at each individual sites Puerto Rico (PR), Prosper, North Dakota, USA (ND), Popayan, Colombia (POP), Citra, FL, USA (CIT), and Palmira, Colombia (PAL).**

|  | CIT | ND | PAL | POP | PR |
| --- | --- | --- | --- | --- | --- |
| RMSE | 5.67 | 25.83 | 2.07 | 7.4 | 2.8 |
| BIAS | -10.64 | 16.79 | -4.77 | 3.37 | -4.01 |
| Adjusted r^2^ | 0.71 | 0.42 | 0.76 | 0.82 | 0.67 |
